# Identification of Optimal Insertion Site in Recombinant Newcastle Disease Virus (rNDV) Vector Expressing Foreign Gene to Enhance Its Anti-Tumor Effect

**DOI:** 10.1371/journal.pone.0164723

**Published:** 2016-10-13

**Authors:** Ziye Pan, Jinjiao He, Lubna M. Rasoul, Yunye Liu, Ruixiang Che, Yun Ding, Xiaocheng Guo, Jiarui Yang, Dehua Zou, Hua Zhang, Deshan Li, Hongwei Cao

**Affiliations:** 1 College of Life Science and Technology, HeiLongJiang BaYi Agricultural University, Daqing, 163319, China; 2 College of Life Science, Northeast Agricultural University, Harbin, 150030, China; Korea University College of Medicine and School of Medicine, REPUBLIC OF KOREA

## Abstract

Recombinant Newcastle disease virus (rNDV) is tumor selective and intrinsically oncolytic, which has been developed as a vector to express exogenous genes to enhance its oncolytic efficacy. Our previous studies found that insertion sites of foreign gene in rNDV vector affected its expression and anti-tumor activities. However, the optimal insertion site for foreign genes remains unknown. In this study, we inserted the enhanced green fluorescence protein (EGFP) and IL2 genes into four different intergenic regions of the rNDV using reverse genetics technology. Recombinants rNDV-EGFPs and rNDV-IL2s were successfully rescued, which displayed the similar growth kinetics with parental virus. Both EGFP mRNA and protein levels were most abundant in HepG2 cells, when EGFP gene was inserted between the NP/P site of the rNDV. Similarly, the IL-2 expressed by HepG2 cells infected with rNDV-IL2 was highest, when IL2 was inserted into NP/P site. To test whether these rNDVs that express higher foreign genes could induce stronger anti-tumor response, we treated the H22-oxter-tumor-bearing C57BL/6J mice with rNDV-IL2s and then examined the oncolytic efficacy. The results showed that rNDV-IL2-NP/P had the strongest inhibition of murine hepatoma carcinoma tumors. The splenocytes isolated from the mice treated with rNDV-IL2-NP/P reached the highest degrees of CD4^+^ T and CD8^+^ T cells. In addition, animals’ survival rate in rNDV-IL2-NP/P-treated group was higher than that of other groups. Taken together, these results demonstrate that NP and P gene junction in rNDV is the optimal insertion site for foreign genes expression to enhance rNDV’s anti-tumor effects.

## Introduction

Newcastle disease virus (NDV), a single negative stranded RNA virus, belongs to member of the *Avulavirus* genus in the *Paramyxoviridae* family. The NDV genome contains six transcriptional units (NP, P, M, F, HN and L), which encode eight proteins in the order NP-P/V/I-M-F-HN-L [1]. The genome of NDV contains a series of non-transcribed intergenic (IG) sequences. These junction regions consist of three elements, which are known as gene-end (GE), IG, and gene-start (GS) sequences [2]. From the 3’ end to the 5’ end, NDV genome is transcribed by a stop-and-restart mechanism at each transcriptional unit, and the expression levels of viral proteins are attenuated from the NP to L protein [3]. Nonstructural protein (V), which acts as an interferon (IFN) α/β antagonist, might contribute to the inherent viral oncolytic properties derived from defective IFN signaling pathways in tumor cells [4]. Normal cells with an effective antiviral response hamper viral replication, thus NDV can replicate up to 10,000-fold more readily in human tumor cells than in normal cells [5]. Several studies show that NDV not only has a typical oncolytic effect, but can also stimulate anti-tumor immunity. Clinical trials for cancer treatment have demonstrated NDV to be well tolerated with few adverse side effects [6]. These events make NDV a promising candidate as a delivery vector for cancer therapy.

NDV grows to very high titers in many tumor cell lines and elicits strong humoral and cellular immune responses in normal cells *in vivo*, which makes this vector an attractive vehicle for the expression of foreign genes [7, 8]. With the development of reverse genetics technology for NDV, the NDV genome can be modified as well as introduced foreign genes to refine its anti-tumor activity. Up to date, cytokines have been found to be effective immunomodulators in animal models and in clinical testing, suggesting that anti-tumor effect is closely related to its expression levels [9]. Among a large number of cytokines, interleukin-2 (IL2), as a strong stimulator of T cells responses, activates natural killer (NK) cells and promotes differentiation of B cells and antibody production. IL2 is the only one approved by the Food and Drug Administration (FDA) for the treatment of cancer [10, 11]. More importantly, many studies proved that recombinant NDVs (rNDVs) expressing IL2 are promising anti-tumor agents [12]. Therefore, IL2 has been demonstrated as a powerful foreign gene for elevating the anti-tumor effects of rNDVs.

During the past decades, the IL2 gene was usually inserted into different intergenic regions of the rNDV genome. Although our primary study implied that the expression level of IL2 was influenced by insertion sites in rNDV, the optimal insertion site for foreign gene expression remains unclear. In this study, we inserted the foreign genes (enhanced green fluorescence protein (EGFP) and IL2) into four different intergenic regions of the rNDVs, respectively. The growth kinetics, the levels of foreign genes expression and oncolytic efficacy of these rescued rNDVs were evaluated *in vitro* and *in vivo*. Our results demonstrate that the region between NP and P gene is an optimal insertion site for foreign gene expression to enhance anti-tumor effects of rNDV.

## Materials and Methods

### Animals and ethics statement

Six- to eight-week old female C57BL/6J mice were purchased from SLAC Company. Animal experiments were carried out in strict accordance with the recommendations in the Guide for the Care and Use of Laboratory Animals of the National Health and Family Planning Commission of the People’s Republic of China. The protocol was approved by the Committee on the Ethics of Animal Experiments of the HeiLongJiang BaYi Agricultural University (Permit Number: 14–0715). We used humane endpoints and euthanize animals with sodium pentobarbital prior to the end of our experiments. The criteria used to determine when the animals should be anesthesia death includes weakness, inability to obtain feed or water, or moribund conditions. The health of the animals was monitored every day to determine any unexpected deaths and the cause of death in these cases. To minimize animal suffering and distress, all experiments were performed under anesthesia. All of animals died with humane euthanasia by administering sodium pentobarbital according to the animal law of the China.

### Cells and virus

Chicken embryo fibroblast cells (DF-1), baby hamster syrian kidney cells (BHK-21), human hepatocarcinoma cells (HepG2), human glioma cells (U251), human lung epithelial carcinoma cells (A549), human cervical carcinoma cells (HeLa) and mouse hepatocarcinoma cells (H22) were cultured in Dulbecco modified Eagle medium (DMEM, Gibco, USA) containing 10% fetal bovine serum (FBS), 1% penicillin/streptomycin, 1% nonessential amino acids, and 1% sodium pyruvate at 37°C under 5% CO_2_. The full-length LaSota cDNA clone (FLC-NDV) was previously generated in our laboratory [13].

### Construction and rescue of rNDV

The EGFP and IL2 genes flanked with gene-star and gene-end were amplified by PCR using the forward primers and the reverse primers (Table 1). These gene fragments were inserted into the NP/P, P/M, M/F and F/HN non-coding region of the FLC-NDV, resulting in recombinant plasmids (prNDV-EGFPs and prNDV-IL2s), respectively. Rescue of these rNDVs was performed by transfecting both the recombinant plasmids and supporting plasmids into BHK-21 cells as described previously [14]. The rescued rNDVs, which were further confirmed by RT-PCR and hemagglutination inhibition (HI) test, were amplified in SPF chicken embryos more than three times, and the rNDVs were harvested, aliquoted and stored at -80°C.

**Table 1:** Premier sequences used for amplification of EGFP and IL2 gene with PCR.

Primer name		Primer sequences
EGFP	F (NP/P)	5’-GGCGCGCCGGACTAGTGCCACCATGGTGAGCAAGGGCGAGGAGCTGTTCACC-3’
	R (NP/P)	5’-CTCTCAGGCCTTCTACCCGTATTTTTTCTTAATTACTTGTACAGCTCGTCCATGCC-3’
	F (P/M)	5’-CCGCGGGCCACCATGGTGAGCAAGGGCGAGGAGCTGTTCACC-3’
	R (P/M)	5’-GTTTAAACCTTCTACCCGTATTTTTTCTTAATTACTTGTACAGCTCGTCCATGCC-3’
	F (M/F)	5’-GGTTAACCACGGGTAGAAGATTGCCACCATGGTGAGCAAGGGCGAGGAGCTGTTCACC-3’
	R (M/F)	5’-CGACGCGTCGTTTTTTCTTACCTCATCTGTGTTTACTTGTACAGCTCGTCCATGCC-3’
	F (F/HN)	5’-GGTTAACCGCCACCATGGTGAGCAAGGGCGAGGAGCTGTTCACC-3’
	R (F/HN)	5’-CGCGTCGCAGTTACTTGTACAGCTCGTCCATGCC-3’
IL2	F (NP/P)	5’-GGCGCGCCCGGACTAGTGCCACCATGTACAGGATGCAACT-3’
	R (NP/P)	5’-GGCCTAGAGGGCCTTCTACCCGTATTTTTTCTTAATCAAGTCAGTGTTGAGATGATG-3’
	F (P/M)	5’-CCGCGGGGACCGCCACCATGTACAGGATGCAACT-3’
	R (P/M)	5’-GTTTAAACCCTTCTACCCGTATTTTTTCTTAATCAAGTCAGTGTTGAGATGATG-3’
	F (M/F)	5’-GTTAACCACGGGTAGAAGATTCCGCCACCATGTACAGGATGCAACT-3’
	R (M/F)	5’-CGACGCGTCGTTTTTTCTTACCTCATCTGTGTTCAAGTCAGTGTTGAGATGATG-3’
	F (F/HN)	5’-GGTTAACCGCCACCATGTACAGGATGCAACT-3’
	R (F/HN)	5’-CGCGTCTCAAGTCAGTGTTGAGATGATG-3’

### Determination of virus titers

The growth characteristics of the rNDVs were evaluated on DF-1 cells in 96-well microplates. The DF-1 cells were infected with rNDV, rNDV-EGFPs or rNDV-IL2s (including different intergenic regions) at a MOI of 10. Every 24 h post infection (hpi), the DF-1 cells were harvested by freeze-thawing three times. Virus titers were determined by 50% tissue culture-infective dose (TCID_50_) for each time point in triplicates from two independent experiments, and log10 values of each mean were charted with standard error.

### Immunofluorescence assay, flow cytometry and quantitative real-time PCR

HepG2, U251, A549, and HeLa cells were mock-infected or infected with rNDV-EGFPs and rNDV-IL2s at a MOI of 10. At 48 hpi, fluorescence images were monitored and photographed using an inverted fluorescence microscope (Nikon, Japan), and the expression levels of EGFP were analyzed by flow cytometry. At the same time, total RNA in infected cells was extracted using Trizol reagent, and quantitative real-time PCR (qRT-PCR) was used to quantify mRNA levels of EGFP and IL2 transcribed from rNDV-EGFPs and rNDV-IL2s using the GeneAmp kit (Applied Biosystems; ABI) [15]. Each qRT-PCR reaction volume (20 μL) contained 10 μL SYBR green real-time PCR master mix (TAKARA), 0.25 μL gene-specific primers (Table 2), and 1 μL standardized template cDNA. All qRT-PCR assays were performed in triplicate in a 96-well plate according to the manufacturer’s protocol. The results were normalized to that of β-actin and expressed as fold changes in relative mRNA expression level using the 2^-ΔΔCT^ method [16].

**Table 2:** Premier sequences used for detecting transcriptional levels of EGFP and IL2 genes with qRT-PCR.

Primer name	Primer sequences
EGFP-F	5’-GCAAAGACCCCAACGAGAAG-3’
EGFP-R	5’-TCACGAACTCCAGCAGGACC-3’
IL2-F	5’-GTGCACCTACTTCAAGTTCTACAAAGA-3’
IL2-R	5’-CATCTGTAAGTCCAGCAGTAAATGC-3’

### Animal experiments

C57BL/6J mice were subcutaneously implanted with H22 cells (5 × 10^6^) in their oxter, and tumors were allowed to grow until the average diameter per mouse reached 4–6 mm. Mice were randomly divided into different groups, and each group included 16 mice. Mice were intratumorally injected with PBS or 1 × 10^7^ pfu of the indicated rNDV-NP/P-IL2, rNDV-P/M-IL2, rNDV-M/F-IL2 and rNDV-F/HN-IL2. The treatments were repeated at one interval day for a total of four sequential times. Tumor volumes were monitored every other day using digital calipers in two dimensions. Tumor volumes are calculated using the following formula: tumor volume (V) = 4/3 × π × S^2^/2 × L/2, where S is the smallest measured diameter and L is the largest diameter. Animals were humanely culled when tumor size reached 18 mm in any dimension or at defined experimental time points. On day 20, all animals in the control group and 6 animals of each treatment group were sacrificed, and their spleens were collected. The remained mice in each group were continually monitored till 120 days with measurement of tumor sizes every other day.

### Determination of lymphocyte populations

Twenty days after tumor transplantation, the mice were sacrificed and the splenocytes were obtained via passing the spleens through 100 μm nylonmesh filters. Lymphocytes were isolated on a density gradient using Lymphoprep^™^ (Sigma-Aldrich, UAS). These cells were then washed once with PBS (pH 7.3) and adjusted to a final concentration of 1 × 10^6^ cells/mL, followed by incubation with mouse monoclonal anti-CD4 conjugated phycoerythrin and anti-CD8 conjugated allophycocyamin antibodies on ice for 2 h in the dark. All cells were finally re-suspended in 300 μL of PBS and analyzed by flow cytometry.

### Statistical analysis

Difference between control and treatment group was compared using SPSS software (Version 19, SPSS-IBM, Chicago, Illinois). The statistical significance of quantitative data was determined with GraphPad prism software (Version 5.01, GraphPad Software Inl., La Jolla, California). The results were expressed as mean ± SEM (standard error of the mean). * *P* < 0.05 and * * *P* < 0.01 were considered statistically significant.

## Results

### Generation of rNDVs and growth kinetics of rNDVs

**Fig 1:**
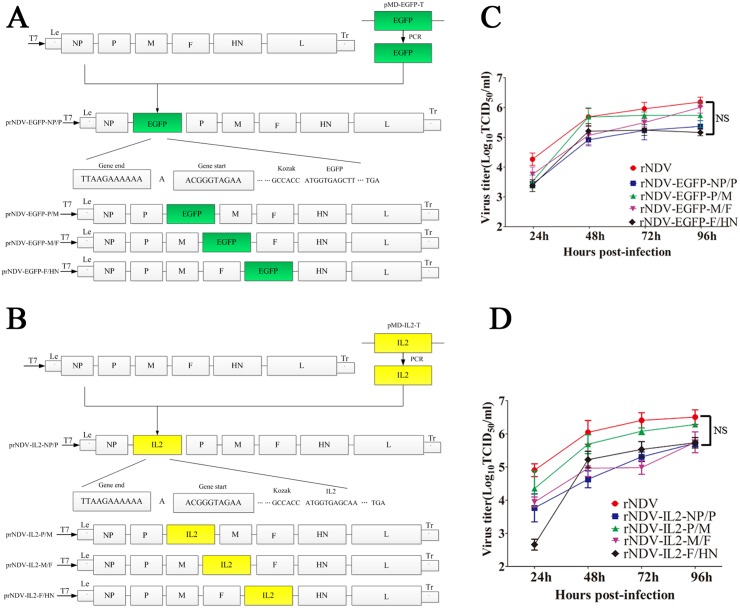
Scheme of the different rNDVs expressing EGFP (A) or IL2 (B) proteins. The small cassette which includes GS, GE, Kozak sequence, and EGFP or IL2 gene was inserted in different intergenic regions in the rNDV genome. The effects of inserted foreign genes on the proliferation of rNDV-EGFPs (C) and rNDV-IL2s (D). Ten-day-old embryonated eggs were inoculated with 100 PFU of the indicated rNDV, and llantoic fluids were harvested at 96 hpi. rNDV titers on DF-1 cells were determined by measuring TCID_50_ and expressed as log10 TCID_50_/mL from three independent experiments (NS: nonsignificant).

Sequencing was performed to confirm that the location and orientation of the inserted foreign genes were correct in the resultant plasmids. The reverse genetics technology was applied to rescue the recombinant virus delivering EGFP (Fig 1A) or IL2 (Fig 1B) genes [17]. After propagation in embryonated eggs for 3 days, the recovered recombinant viruses exhibited similar biological phenotypes of their parental natural isolates. Meanwhile, growth characteristics of recombinant viruses were examined in a single-step growth cycle in DF-1 cells to compare the growth characteristics of the rNDVs. The data showed that the growth kinetics of rNDV-EGFPs (Fig 1C) and rNDV-IL2s (Fig 1D) were slightly delayed and eventually can reach to the similar levels while compared with the parental virus LaSota strain. These results suggest that there isn’t significant difference between rNDVs and parental virus in growth kinetics.

### The expression levels of EGFP by rNDV-EGFPs

**Fig 2:**
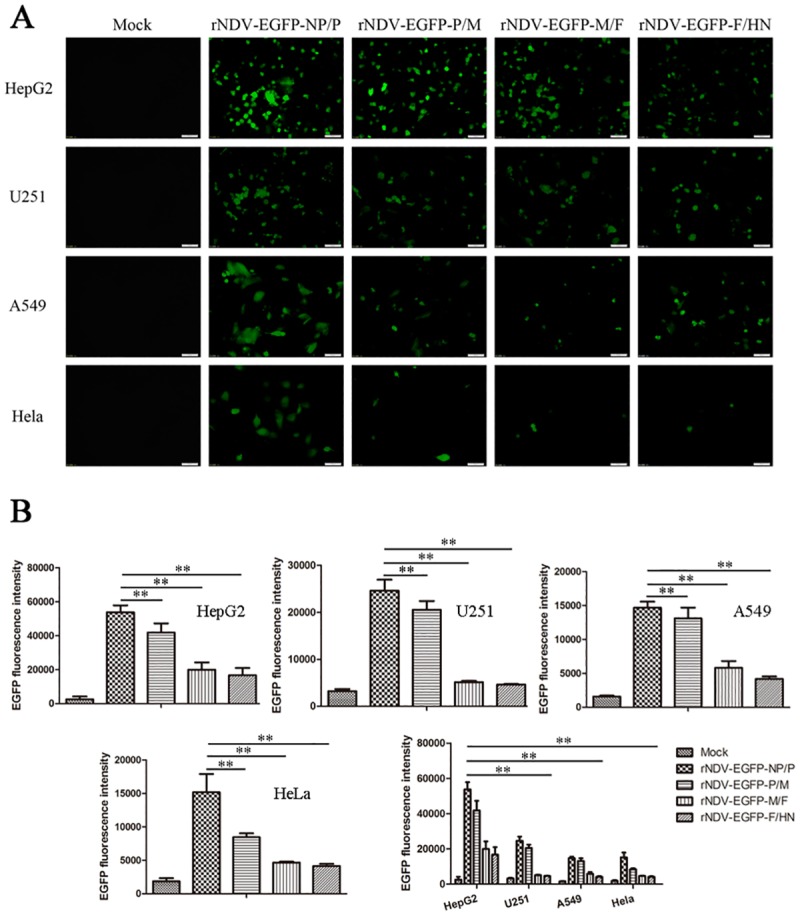
(A) Rescued viruses stably express EGFP in different tumor cells. rNDV-EGFPs at MOI of 10 were used to infect tumor cells in 6-well plate. At 48 hpi, the fluorescence in the infected cells were detected and digitally photographed by fluorescence microscopy. The white bar represents 20 μm. (B) Measurement of EGFP fluorescence intensity. Tumor cells in 6-well plate were mock-infected or infected with rNDV-EGFPs at MOI of 10 for 48 h, and EGFP fluorescence intensity were analyzed using flow cytometry. The results were expressed as the quantity of the mean EGFP fluorescence cells with SEM (* *P* < 0.05; ** *P* < 0.01).

The expression levels of EGFP in the HepG2, U251, A549 and HeLa cells infected with rNDV-EGFPs at MOI of 10 were detected by fluorescence microscopy, respectively. At 48 hpi, green fluorescence could be readily observed in these cells infected with rNDV-EGFPs except for the mock-infected cells (Fig 2A). Fluorescence intensity in tumor cells generated by the rNDVs was gradually diminished in the order of NP/P, P/M, M/F and F/HN junctions. These results proved that rNDVs not only were rescued successfully, but also could express foreign gene correctly. In order to further confirm that the expression levels of EGFP decreased regarding to the insertion position toward the 5’ end of the viral genome, we used flow cytometry to analyze fluorescence intensity in tumor cells infected with rNDV-EGFPs. The results showed that HepG2 cells infected with rNDV-EGFP-NP/P displayed highest green fluorescence intensity compared with other cell lines (Fig 2B). The fluorescence intensity of EGFP was gradually decreased from NP/P to F/HN expression site, suggesting that the resultant decrease in EGFP expression was likely a reflection of a transcription gradient by the viral RNA-polymerase.

### The transcriptional levels of foreign genes at different intergenic positions of rNDV

**Fig 3:**
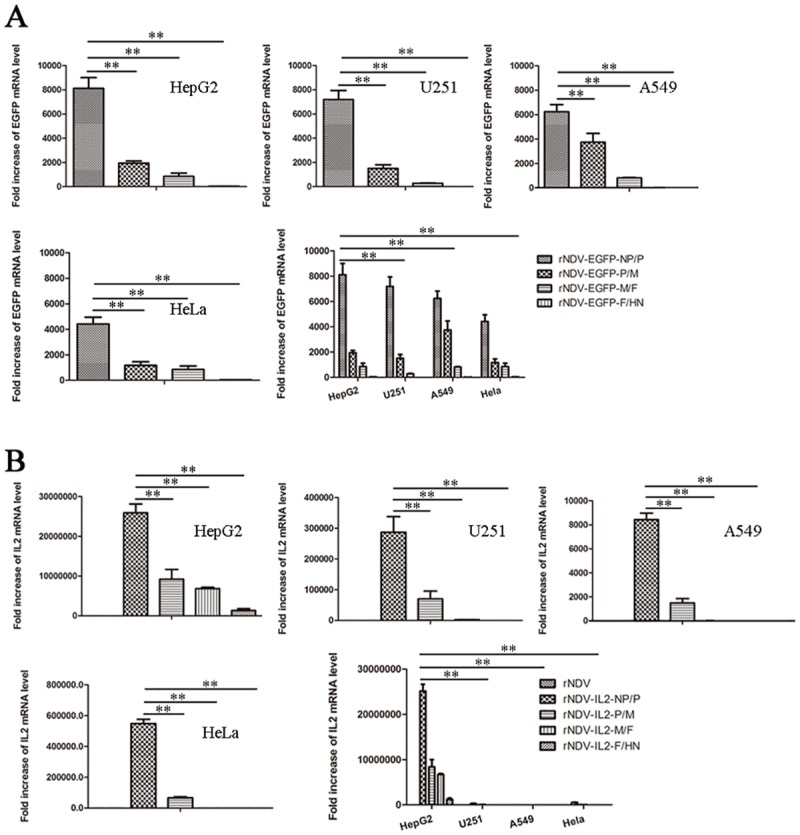
Quantification of the mRNA transcriptional levels of EGFP (A) and IL2 (B). Tumor cells in 6-well plate were infected with the indicated rNDV-EGFPs or rNDV-IL2s at MOI of 10. Total RNA in infected cells was extracted using Trizol reagent at 48 hpi, and qRT-PCR was performed to quantify mRNA transcriptional levels of EGFP and IL2, respectively. Transcriptional levels of EGFP and IL2 genes are calculated relative to the β-actin, and the data are expressed as fold changes with SEM (* *P* < 0.05; ** *P* < 0.01).

To evaluate the transcriptional levels of foreign genes introduced into rNDVs, mRNA levels of EGFP and IL2 in HepG2, U251, A549 and HeLa cells were analyzed by qRT- PCR. The results showed that the EGFP (Fig 3A) and IL2 (Fig 3B) mRNA were most abundance when we inserted these genes into NP/P junction. The EGFP and IL2 mRNA abundance gradually decreased dependent on the insertion position toward form 3’ end to 5’ end of the viral genome. These results proved that mRNA levels of foreign genes were affected by intergenic regions in rNDV. Furthermore, the transcriptional level of foreign gene in HepG2 cells was higher than that of other tumor cells (P < 0.01), suggesting that expression of foreign genes is extremely highest in human hepatocarcinoma cells, when they were inserted into NP and P gene junction.

### rNDVs inhibited tumor growth and enhanced animals’ survival rate

**Fig 4:**
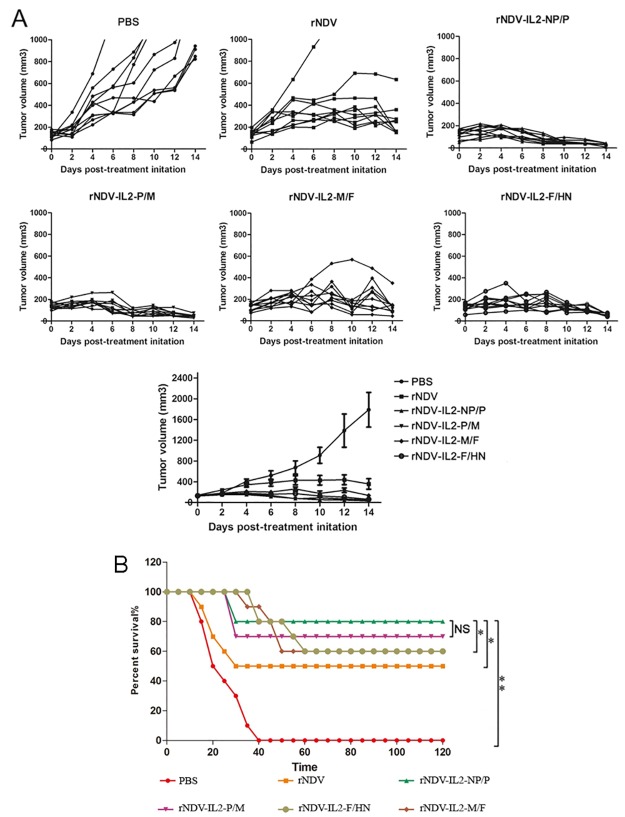
(A) rNDV/IL2s effectively suppressed tumor growth. H22 oxter tumor-bearing mice model was treated with rNDV/IL2s, rNDV and PBS, and tumor volume was measured every other day using digital calipers in two dimensions. (B) Survival of H22 models animal in 120 days period after treatment with the rNDV-IL2s, rNDV, and PBS. The tumor-bearing mice were sacrificed when the tumor volume grew to a significant size (diameter > 18 mm). All the values are the mean and SEM of 10 samples. The log-rank test reveals a significant effect (NS: nonsignificant; * *P* < 0.05; ** *P* < 0.01).

In order to evaluate anti-tumor efficacy conferred by rNDV-IL2 *in vivo*, H22-oxter-tumor-bearing mice model described earlier [13] were treated with rNDVs, and their tumor volumes were measured every other day. During the period of treatment, there were scarcely side effects except slight swelling at the injection site. Mice treated with rNDV-IL2 showed a reduction in tumor volume compared with the PBS- and rNDV-treated groups (P < 0.01) (Fig 4A), suggesting that IL2 significantly enhanced the anti-tumor effects of rNDV. In addition, PBS-treated mice began to succumb to tumor progression (tumor diameter > 18 mm) on day 15, and they can survive till day 42. Meanwhile, mice treated with rNDV showed a significant reduction of tumors, with 5 of 10 mice undergoing complete regression in HCC models compared with 0 of 10 mice in the PBS-treated group during the 120 days of observation (Fig 4B). In contrast, less than 5 of the10 animals in rNDV-IL2 groups developed several signs of clinical tumor symptom. Only 2 of the 10 animals in rNDV-IL2-NP/P group died by the end of animal experiment, indicating that rNDV expressing IL2 i*n vivo* notably enhanced animals’ survival rate.

### Mice treated with rNDVs resulted in immune cell accumulation

**Fig 5:**
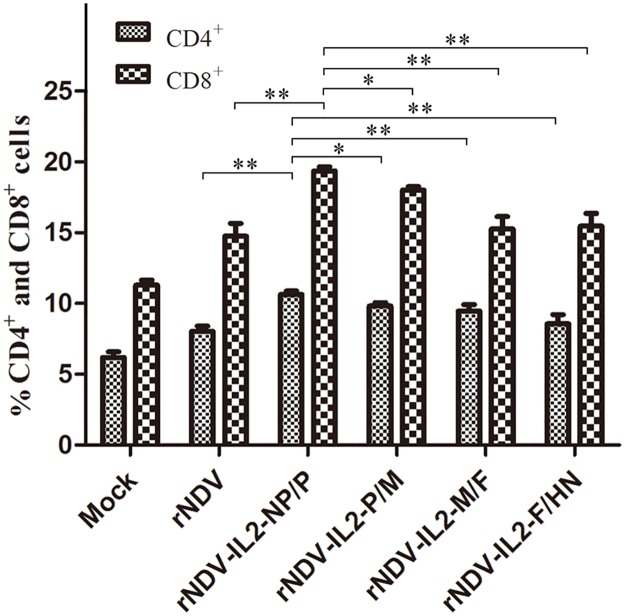
Percentage of the CD4^+^ T and CD8^+^ T cells in spleen from H22 models mice treated with rNDV-IL2s. The CD4^+^ T and CD8^+^ T cells isolated from spleen of H22 models mice mock-treated or treated with rNDV-IL2s or rNDV were analyzed by flow cytometry (* *P* < 0.05; ** *P* < 0.01).

Combination of the above anti-tumor effects of rNDVs in *vivo*, we further analyzed percentage of CD4^+^ T and CD8^+^ T cells from mice treated with rNDVs after 12 days. The results showed that the percentage of both CD4^+^ T and CD8^+^ T cells in each rNDV-IL2 groups significantly increased when compared with mock- and rNDV-treated groups, suggesting that inserting IL2 gene in rNDV could extremely enhanced the numbers of CD4^+^ T and CD8^+^ T cells (Fig 5). Moreover, the mice treated with rNDV-IL2-NP/P exhibited the highest percentage of CD4^+^ T (11.1%) and CD8^+^ T cells (20%) among the rNDV-IL2-treated groups, revealing that P and M gene junction is the optimal insertion site for foreign gene expression and thus can elevate the T cells immune response.

## Discussion

rNDV has a number of advantages as viral vectors for a broad range of different cancer therapy. Its genome can stably express foreign gene; and the expression level of a foreign gene can be affected by the insertion region [18, 19]. However, the optimal insertion site for foreign gene expression to increase rNDV’s anti-tumor efficacy remains elucidated. In this study, we inserted EGFP and IL2 genes into rNDV genome at four different gene junctions, and determined the expression levels of foreign genes and their oncolytic activity in tumor-bearing mice model. This is the first study to demonstrate that the non-coding region between the NP and P gene junction is an optimal insertion site for foreign gene expression to enhance the anti-tumor effects of rNDV.

It is well known that NDV mRNA transcriptional level is gradually decreased from the 3’ end to 5’ end of the viral genome [20]. Similarly, whether the expression level of foreign gene inserted into viral genome will decrease in accordance with mRNA levels remains to be elucidated. Based on the encouraging previous studies that inserting foreign gene elevates effectiveness of rNDV against tumor while it is safety in normal tissues [21], we began to investigate the underlying mechanism behind rNDV’s oncolytic effect enhanced by foreign gene. In this present study, the EGFP and IL2 genes flanked by NDV transcription gene-start and gene-end sequences were inserted into NP/P, P/M, M/F and F/HN junctions, respectively. These recombinant viruses that were introduced foreign genes were successfully rescued using reverse genetics technology. Growth kinetics of the rNDVs was not distinguishable from that of the parent virus, which indicated that the insertion of the EGFP or IL2 in the genome had less effect on the viral life cycles. Our immunofluorescence assay demonstrated that EGFP expression was gradually decreased when inserted from NP/P to F/HN expressing sites. Flow cytometry and qRT-PCT assays consistently demonstrated that quantity of the EGFP fluorescence intensity in tumor cells infected with rNDV decreased following the order from 3’ end to 5’ end of viral genome. Foreign gene mRNA was most abundance when inserted into NP/P junction, suggesting that foreign gene was inserted closer to 5’ end of the rNDV viral genome, whose transcriptional and expression levels were relatively higher. Therefore, our findings support that the transcriptional levels of viral mRNA are attenuated from the NP to L genes [3]. In addition, we found that the transcriptional and expression levels of foreign gene in rNDVs-infected HepG2 cells were higher than that of other tumor cell lines. These results implied that foreign gene inserted in the non-coding region between the NP and P genes could be very useful for future therapeutic hepatoma carcinoma.

Our previous studies found that LaSota/IL2 has a potential immunotherapy and oncolytic therapy for cancers [12], but the optimal insertion site for IL2 expression remains to be further investigated. Therefore, we used H22 oxter tumor-bearing mice model to evaluate expression levels of IL2 and its anti-tumor efficacy *in vivo*. The results indicated that rNDV/IL2s significantly inhibited tumor growth with more than 92% inhibition rate compared with rNDV- and PBS-treated groups [18]. Although there was no significant difference, mice treated with rNDV-IL2-NP/P had a little higher tumor regression and exhibited higher survival rate than the other rNDV-treated groups. Combination of the above results in cell experiment, we speculated that successfully expressed IL2 might activate and increase tumor infiltration with CD4^+^ T and CD8^+^ T cells for tumor regression. These findings promoted us to further investigate whether IL2 expression levels *in vivo* had any contribution to CD4^+^ T and CD8^+^ T cells activated in tumor-bearing mice model. Hence, we analyzed the splenocytes isolated from rNDV-treated tumor-bearing mice, and found that the percentage of CD4^+^ T and CD8^+^ T cells in rNDV-IL2-NP/P treated group were much higher than other rNDV-treated groups. These data demonstrate that the region between NP and P gene is probably an optimal insertion site for IL2 expression to enhance anti-tumor effects of rNDV.

In conclusion, we have successfully constructed eight recombinant viruses vectoring EGFP and IL-2 at different intergenic regions (NP/P, P/M, M/F, and F/HN). Our study showed that both EGFP and IL-2 were expressed most abundant in HepG2 cells, when foreign genes were inserted between the NP/P site of the rNDV. In addition, rNDV-IL2-NP/P had the strongest inhibition of murine hepatoma carcinoma tumors growth, elicited highest degrees of splenocytes CD4^+^ T and CD8^+^ T cells, and obviously enhanced animals’ survival rate. Overall data demonstrate that the junction between NP and P genes is an optimal insertion site for foreign gene expression for cancer therapy.
