# Correction: Identification of Optimal Insertion Site in Recombinant Newcastle Disease Virus (rNDV) Vector Expressing Foreign Gene to Enhance Its Anti-Tumor Effect

**DOI:** 10.1371/journal.pone.0171400

**Published:** 2017-01-30

**Authors:** Ziye Pan, Jinjiao He, Lubna M. Rasoul, Yunye Liu, Ruixiang Che, Yun Ding, Xiaocheng Guo, Jiarui Yang, Dehua Zou, Hua Zhang, Deshan Li, Hongwei Cao

The affiliation for the third author is incomplete. Lubna M. Rasou is affiliated with: College of Life Science, Northeast Agricultural University, Harbin, 150030, China and College of Science, University of Baghdad, Department of Biology, Baghdad 10071, Iraq.

## References

[1] Pan Z, He J, Rasoul LM, Liu Y, Che R, Ding Y, et al (2016) Identification of Optimal Insertion Site in Recombinant Newcastle Disease Virus (rNDV) Vector Expressing Foreign Gene to Enhance Its Anti-Tumor Effect. PLoS ONE 11(10): e0164723 doi:10.1371/journal.pone.0164723 27736965 10.1371/journal.pone.0164723 PMC5087999

